# Tumorigenesis Caused by Aberrant Expression of GANP, a Central Component in the Mammalian TREX-2 Complex—Lessons from Transcription-Coupled DNA Damages

**DOI:** 10.3390/ijms252413612

**Published:** 2024-12-19

**Authors:** Andri Rezano, Naomi Gondo, Yasuhiro Sakai, Yuko Nakamura, Suchada Phimsen, Tokio Tani, Akihiko Ito, Seiji Okada, Kazuhiko Kuwahara

**Affiliations:** 1 Department of Biomedical Sciences, Division of Cell Biology, Faculty of Medicine, Universitas Padjadjaran, Sumedang 45363, West Java, Indonesia; andri.rezano@unpad.ac.id; 2 Department of Breast and Thyroid Surgical Oncology, Sagara Hospital, Kagoshima 892-0833, Kagoshima, Japan; ngondo@sagara.or.jp; 3 Department of Tumor Pathology, Hamamatsu University School of Medicine, Hamamatsu 431-3192, Shizuoka, Japan; ya-sakai@hama-med.ac.jp; 4 Department of Diagnostic Pathology, Kindai University Hospital, Osaka-sayama 589-8511, Osaka, Japan; y.nakamura@med.kindai.ac.jp; 5 Department of Biochemistry, Faculty of Medical Science, Naresuan University, Phitsanulok 65000, Thailand; suchadaph@nu.ac.th; 6 International Research Center for Agricultural and Environmental Biology (IRCAEB), Kumamoto University, Kumamoto 860-8555, Kumamoto, Japan; ttani@kumamoto-u.ac.jp; 7 Department of Pathology, Kindai University Faculty of Medicine, Osaka-sayama 589-8511, Osaka, Japan; aito@med.kindai.ac.jp; 8 Division of Hematopoiesis, Joint Research Center for Retroviral Infection, Kumamoto University, Kumamoto 860-0811, Kumamoto, Japan; okadas@kumamoto-u.ac.jp; 9 Department of Diagnostic Pathology and Genome Medical Center, Kindai University Hospital, Osaka-sayama 589-8511, Osaka, Japan

**Keywords:** cancer, chemosensitivity, GANP, transcription-coupled DNA damage, TREX-2 complex

## Abstract

DNA is frequently damaged by genotoxic stresses such as ionizing radiation, reactive oxygen species, and nitrogen species. DNA damage is a key contributor to cancer initiation and progression, and thus the precise and timely repair of these harmful lesions is required. Recent studies revealed transcription as a source of genome instability, and transcription-coupled DNA damage has been a focus in cancer research. Impaired mRNA export is closely related to DNA damage through R-loop formation. The molecular machineries of transcription-coupled DNA damage have been extensively analyzed in *Saccharomyces cerevisiae*. However, the molecular basis of these phenomena in higher eukaryotes remains elusive. In this review, we focus on the relationship between deregulated mRNA export through the transcription-export-2 (TREX-2) complex and cancer development. Particularly, the expression of germinal center-associated nuclear protein (GANP), a molecular scaffold in the TREX-2 complex, is highly associated with tumorigenesis in mice and humans. Although the deregulated expression of other components in the TREX-2 complex might affect cancer development, we have directly demonstrated the significance of GANP in tumorigenesis using genetically modified mice. Additionally, we describe recent evidence for medical applications demonstrating that the downregulation of the other components may be a good candidate for a chemotherapeutic target in terms of reducing the side effects.

## 1. Introduction

Tumorigenesis is a multistep process that leads to the transformation of cells and the acquirement of malignant potential [1]. The continual accumulation of genomic mutations is a key contributor to the transformation process. These mutations can be the result of DNA damage from exogenous genotoxic stresses, such as ultraviolet or ionizing radiation and carcinogenic chemicals. Cancer-promoting mutations can also arise from aberrations in cellular processes, such as DNA replication errors and errors in DNA double-strand break (DSB) repair [2]. DNA can also be damaged during the export of mRNA from the nucleus to the cytoplasm [3]. When mRNA export is hindered, the mRNAs hybridize with one strand of double-stranded DNA in the nucleus to form a structure known as an R-loop. This structure results in genomic instability and breaks in the DNA, which is called transcription-coupled DNA damage.

The transcription-export-2 (TREX-2) complex is a critical molecular complex that links gene expression with the nuclear mRNA export process. The TREX-2 complex consists of several molecules, including germinal center-associated nuclear protein (GANP), PCI domain containing 2 (PCID2), enhancer of yellow homolog 2 (ENY-2), centrin2/3, and deleted in split-hand/split-foot 1 (DSS1) [4]. Each of these components also has unique functions in addition to an involvement in mRNA export. Studies have shown that each component of the TREX-2 complex is aberrantly expressed in various tumors. The defective function of the TREX-2 complex has been shown to augment the sensitivity of cancer cells towards chemotherapy [5].

In this review, we focus on the relationship between deregulated mRNA export and cancer development, especially through the TREX-2 complex. We also describe recent evidence for medical applications showing that the downregulation of the other components may be a good candidate for chemotherapeutic targets in terms of reducing the adverse effects.

## 2. Overview of the TREX-2 Complex in Yeast and Its Regulation of mRNA Export and Homologous Recombination

mRNA nuclear export machinery has been extensively clarified over recent decades, especially in *Saccharomyces cerevisiae*. Hurt and colleagues identified several molecules that were involved in mRNA export by proteomics techniques and reported that Sac3 was stably associated with Thp1, which was critical for transcription elongation [6]. Sac3 was initially discovered as a complementary gene in a *S. cerevisiae act1-1* mutant [7]. Sac3 is localized in the nucleus, and yeast deficient for Sac3 showed mitotic delay and chromosome instability [8]. The molecular functions of Sac3 have remained unknown for over a decade. Hurt’s group demonstrated that *sac3*- and *thp1*-deficient yeast showed nuclear accumulation of mRNAs, suggesting that these two molecules are crucial for mRNA nuclear export [6]. Both *sac3*- and *thp1*-deficienct yeast had a non-lethal phenotype. The authors subsequently identified additional molecules by proteomics, including Sus1, a component of the SAGA complex with histone acetylase activity, and found that Sus1 interacts with the Sac3-Thp1 complex [9]. Other identified molecules included Cdc31 and Sem1, a yeast centrin and a component of the proteasome complex, respectively. Yeast deficient of each component showed impairments of mRNA nuclear export, suggesting that all components were indispensable for mRNA nuclear export. The complex consisting of these molecules was designated as the transcription-export-2 (TREX-2) complex.

**Figure 1:**
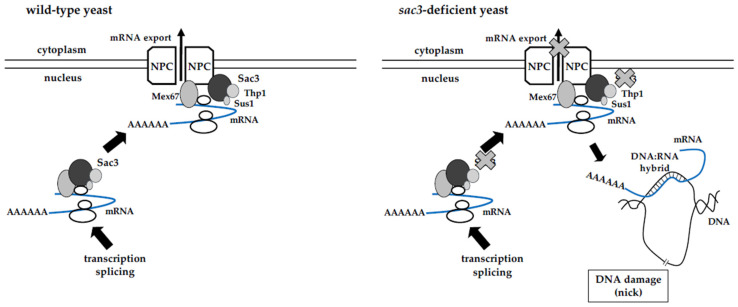
Model for R-loop formation in *sac3*-deficient yeast. Eukaryotic gene expression is regulated by multiple steps. Nascent RNAs (indicated by blue lines) are processed in the nucleus, and then exported to the cytoplasm through the nuclear pore complex. TREX-2 complex, which is critical for mRNA export, is tightly associated with nuclear pore complex. Deficiency of any component in the TREX-2 complex (in this case, *sac3*-deficiency) results in the impairment of mRNA export. Accumulated mRNAs in the nucleus hybridize to the template DNA strand and form DNA:RNA hybrid. The non-template DNA is separated as single-strand DNA which is susceptible to DNA damages.

Aguilera and colleagues identified the Sac3-Thp1 complex around the same time. The authors found that several *S. cerevisiae* mutants exhibited hyper-recombination phenotypes using an artificial recombination substrate composed of a truncated *leucine*-tandem-repeat construct; after a recombination process, functional leucine is produced, which is essential for yeast survival. Both *sac3*-deficient and *thp1*-deficient yeast showed a similar hyper-recombination phenotype [10,11]. Importantly, Aguilera proposed a DNA:RNA hybrid model that links transcription with recombination during mRNA metabolism [12]. Upon the disruption of mRNA export, mRNA accumulates in the nucleus and anneals with a single-stranded DNA template to form a DNA:RNA hybrid, which disturbs the other DNA strand and leads to a dispersed single DNA strand. This unique structure of the DNA:RNA hybrid and the dispersed single DNA strand, the so-called R-loop, is susceptible to DNA breaks, resulting in genomic instability. These DNA breaks are defined as transcription-coupled DNA damage, and repair is presumably mediated by homologous recombination (HR) (Figure 1).

## 3. Structural Features of GANP, a Central Component in the TREX-2 Complex

**Figure 2:**
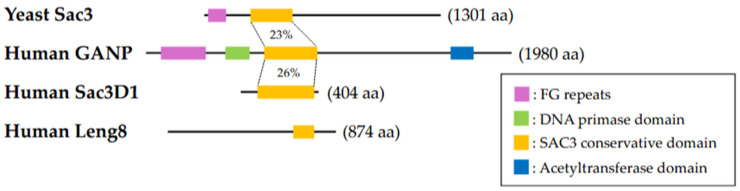
Schematic diagram of Sac3/GANP family proteins. Mammalian GANP consists of several domains including phenylalanine–glycine (FG) repeats, a DNA primase domain, a Sac3 conservative domain, and an acetyltransferase domain. Sac3, a yeast orthologue of GANP, has only a Sac3 conservative domain, suggesting that GANP bears additional functions compared to Sac3. Although SAC3D1 and LENG8 belong to the Sac3/GANP family, LENG8 has half of a Sac3 conservative domain.

As described above, the human counterparts of the yeast TREX-2 complex have been identified, including GANP, PCID2, ENY-2, centrin2/3, and DSS1 (the orthologues of *S. cerevisiae* Sac3, Thp1, Sus1, Cdc31, and Sem1, respectively). The depletion of TREX-2 eliminates the connection of transcribed genes to the nuclear pore complex. TREX-2 is thus implicated as a crucial factor linking the mRNP export complexes in the nuclear interior to nuclear pore complexes. GANP was initially identified in a study searching for a novel molecule involved in mouse B-cell maturation and differentiation. A monoclonal antibody, 29-15, that recognized an upregulated molecule in germinal centers was established. Using an expression cloning method with a λgt11 phage library, a novel mouse gene was identified, designated as *germinal center-associated nuclear protein* (*ganp*), encoding a 210 kDa nuclear protein composed of 1971 amino acids known as GANP. Further study revealed that the middle portion of GANP (approximately 600 amino acids) is homologous with that of the *S. cerevisiae* Sac3 protein sequence (23% at the amino acid level) (Figure 2), and this region is evolutionally conserved among several species (*Schizosaccharomyces pombe*, *Caenorhabditis elegans*, *Drosophila melanogaster*, and *Xenopus laevis*), indicating that GANP is the mammalian counterpart to yeast Sac3 [13,14]. This region was also observed in Sac3D1 [15] and Leng8, and thus we propose these proteins as Sac3/GANP family members.

The carboxyl-terminal portion of mouse GANP is highly homologous to human MCM3-acetylating protein (MCM3AP; former name, Map80) [16]. MCM3AP binds to and acetylates MCM3, which is essential for DNA replication [17]. A comparison of the isolated human *ganp* from the chromosome 21q22.3 locus and this carboxyl-terminal portion showed that this region was identical to human *mcm3ap*. However, the relationship between GANP and MCM3AP is still controversial. Takei and Tsujimoto reported an 80 kDa band in the immunoblotting of HeLa cell extracts with an antibody recognizing the carboxy-terminal of human GANP [16]. In contrast, Northern blot analysis using *ganp*- and *mcm3ap*-specific probes revealed only a 6 kb band, and no shorter band was detected [14], suggesting that MCM3AP might be an alternative spliced form of GANP or a shorter form of GANP with post-translational modification. Another group demonstrated the existence of a unique promoter in human *mcm3ap* transcription [18]. Mouse MCM3AP has not yet been reported, because the specific antibody recognizing the corresponding region is not available. Using this type of antibody, it might be clarified whether both 220 kDa and 80 kDa bands appear in immunoblot analysis. Therefore, the functions of mouse MCM3AP in vivo remain elusive, although it has MCM3-binding/acetylating domains.

A 150 amino acid sequence in the amino-terminal region of GANP shows significant similarity to the DNA primase p49 [19]. The amino-terminal region of GANP also contains FG repeats that bind to the nuclear RNA export factor 1 (NXF1) FG-binding domain, suggesting GANP’s involvement in NXF1-containing mRNP transfer to the nuclear pore complex [20]. Lower species lack some functional domains and harbor FG-repeat and the Sac3 conserved domains (Figure 2).

## 4. HR and R-Loop

DNA DSBs caused by cytotoxic chemicals or ionizing radiation are precisely repaired by HR, which is a multistep DNA repair mechanism mediated by more than 20 molecules [21]. DSBs are the most harmful lesion and are precisely repaired by HR, nonhomologous end joining, and other alternative pathways such as microhomology-mediated end joining and single-strand annealing. The choice of repair pathway depends on the cell cycle stage and cell type. In contrast to nonhomologous end joining, microhomology-mediated end joining, and single-strand annealing, HR requires intact DNA templates for more accurate repair. Several models of DNA repair by HR have been reported.

The most upstream molecules in the HR pathway are ataxia telangiectasia-mutated (ATM) and ataxia telangiectasia and Rad3-related (ATR), which mediate the phosphorylation of downstream effectors, including p53, the partner and localizer of BRCA2 (PALB2), and RAD51, as well as histone H2AX, which acts as a marker for DSBs and triggers the recruitment of chromatin remodelers and other end resection factors. PALB2 recruits breast cancer susceptibility gene 2 (BRCA2) and RAD51 to single-stranded DNA. ATM and ATR also phosphorylate checkpoint kinases CHK1 and CHK2, thus orchestrating signaling events that lead to checkpoint arrest while activating the machinery involved in HR [22,23].

As previously described, the impairment of mRNA biogenesis induced R-loop formation; however, this is also observed under physiological conditions like immunoglobulin class-switch recombination [24]. In some cases, R-loops may be necessary for the control of gene expression. Experimental evidence shows that the accumulation of R-loop leads to harmful effects like genomic instability; therefore, accumulated R-loops should be avoided. One mechanism is caused by the resolution of negative supercoiling by topoisomerase 1, supporting that an R-loop is accumulated by the treatment of topoisomerase inhibitors [25]. Another mechanism is presumably to remove the R-loop by RNase H and RNA-DNA helicases [26]. According to transcriptional blocks, R-loop is known as a source of DNA damage. The dissolution of R-loops may be dependent on members of Fanconi anemia (FA) factors including BRCA1 and BRCA2. Transcription-coupled DNA damage is presumably repaired by HR. Whether the molecules associated with the repair of transcription-coupled DNA damage are identical or similar to ones in classical HR pathways is unknown.

In human breast cancer cell lines, it is reported that R-loop accumulation is frequently observed at such estrogen-induced genes [27]. This suggests that DNA damage may be induced by R-loops around active genes.

## 5. The Regulation of Recombination and mRNA Nuclear Export by the Mammalian TREX-2 Complex

In the event of transcription-coupled DNA damage, the damage should be repaired prior to the G1/S checkpoint. In yeast deficient for any component of the TREX/THO or TREX-2 complexes, genomic damage occurs. THO is a multimeric complex required for mRNA-protein biogenesis [28]. Defects in TREX-2 lead to aberrations in replication or survival, signifying the importance of the function of individual components in the TREX-2 complex.

To examine whether TREX-2 orthologues in mammals have similar functions to those in *S. cerevisiae*, a *β-galactosidase* tandem-repeat construct was established to quantitatively measure the cell recombination rate [29]. Using a tandem *β-galactosidase* reporter system, researchers found that GANP suppresses homology-mediated DNA recombination, particularly in rapidly proliferating cells or cells with high-grade DSBs, as in germinal center B cells, which is required for DNA repair. This regulatory effect on DNA recombination depended on the Sac-3 conserved region but was also affected by the C-terminal region of GANP, which contains a HAT domain [29]. Thus, GANP might play distinct roles in DNA replication and repair mechanisms.

This sensor mechanism detects DNA damage during cell proliferation, thus regulating the cell cycle. GANP and PCID2 selectively regulate mitotic spindle checkpoints Shugoshin-1 and MAD2 to maintain the cellular genome, presumably through mRNA export machinery [30,31]. DSS1 plays a role in BRCA2 stabilization by regulating its ubiquitin-dependent proteolytic degradation [32]. This suggests a unique function for DSS1 in the organization of ribonucleoprotein complexes during transcription, nuclear to cytoplasmic export, and translation. Unlike GANP and PCID2, whether DSS1 is involved in specific mRNA(s) export is unclear.

The functional and structural characterization of the mammalian TREX-2 complex has demonstrated how the complex links transcription/processing with nuclear mRNA export. A portion of TREX-2 was found to be located near the transcription site in the mammalian nucleus. After maturing into a transport-competent state, the mRNP particle was proposed to bind with TREX-2 through the interaction of GANP with NXF1, facilitating its delivery to the nuclear pore complex [33]. Apart from the yeast TREX-2 complex, the mRNA export mechanism through the mammalian TREX-2 complex seems more complicated in terms of specific mRNA export [30].

Genome maintenance has been shown to be regulated by proteins involved in DNA replication or DNA damage response. Subsequent studies revealed that mutations in genes involved in pre-mRNA splicing and the biogenesis and export of mRNAs result in DNA damage and genome instability. The instability is frequently mediated by R-loops formed by DNA-RNA hybrids and displaced ssDNA. Mitotic HR protects the cellular genome by properly repairing transcription-coupled DNA damage and other lesions caused by exogenous insults. During proliferative stages in development and somatic cell renewal in adults, it is essential to repair DNA damage and protect cells against cell death and mutagenic outcomes [34]. In yeast, HR primarily involves proteins within the RAD52 group. In mammalian cells, the BRCA2 tumor suppressor protein plays a central function in HR by regulating RAD51-binding DNA, which is required for DSB repair. BRCA2 forms a complex with DSS1 to promote the RAD51-loading activity of BRCA2. The binding of DSS1 masks the nuclear export signals of BRCA2 and regulates both BRCA2 and RAD51 nuclear localization [35]. DSS1 was also shown to promote BRCA2-dependent HR by targeting replication protein A (RPA). DSS1 is thought to mimic DNA and reduce the affinity of RPA for ssDNA [36].

Some studies reported that R-loops were not detected in TREX-2-depleted cells, but R-loop accumulation was observed in BRCA2-depleted cells [37]. BRCA2 was shown to be associated with PCID2, which prevents R-loop formation and transcription-coupled DNA damage. These results indicate that BRCA2 is directly or indirectly required for R-loop formation and presumably transcription-coupled DNA damage, leading to carcinogenesis [37].

## 6. Functional Role of Other TREX-2 Components Except for GANP in Tumor Development

Studies in mice with conditionally targeted PCID2 in B cells demonstrated that PCID2 is essential for B-cell development, presumably through deregulated MAD2 [32]. PCID2 was also reported to be associated with E1A-like inhibitor of differentiation one (EID1), which is involved in stemness in embryonic stem and induced pluripotent stem cells [38]. EID1 inhibits the HAT activity of CBP/p300 to suppress developmental gene expression. Whether TREX-2 components are functionally related to embryonic stem cells to sustain their pluripotency is still unknown. Whey acid protein (wap)-cre-pcid2-deficient mice were generated, but no spontaneously developed mammary tumors were observed over a 2-year observation (unpublished observation). Whether the aberrant expression of PCID2 is associated with mammary tumorigenesis is unknown. Rather, the unique pathway involved in mammary tumorigenesis might exist downstream of GANP.

The relationship of PCID2 with gastrointestinal tumors is more concrete, and the underlying pathway is not related to TREX-2. Clinical studies have shown that the level of *PCID2* mRNA was significantly higher in colorectal cancer (CRC) tissues than in adjacent normal tissue, and its high expression was associated with recurrence [39]. PCID2 may accelerate the G1-S cell cycle transition and inhibit apoptosis in CRC cells. The upregulation of cyclin D1 and the downregulation of p21Cip1 are linked to the PCID2-induced G1-S transition [40]. The aberrant expression of PCID2 also contributes to the degradation of the tumor suppressor promyelocytic leukemia (PML) through ubiquitin proteasome system-dependent degradation [41]. Under normal conditions, PML suppresses oncogenic Wnt/β-catenin signaling while enhancing tumor suppressive β-catenin signaling. If PML is suppressed, such as in the case of aberrant PCID2 expression, oncogenic Wnt/β-catenin signaling is activated, thus contributing to CRC tumorigenesis. When cells with elevated PCID2 expression were implanted in an in vivo model, an increased tumor weight and incidence of metastasis were observed, which further proved that PCID2 is an important factor in the tumorigenesis and progression of CRC [39].

Other TREX-2 components like ENY-2, which is associated with deubiquitination machinery, are also markedly elevated in many types of cancers including hepatocellular carcinoma and ovarian cancer. The deregulation of mRNA export by ENY-2 and cell cycle disruption is a key to the development of cancers. Additionally, ENY-2 may facilitate immune evasion by modifying and disrupting the balance between MHC, immune suppressors, and immune stimulators [42]. In head and neck squamous cell carcinoma, the overexpression of ENY-2 is correlated with poor prognosis [42], which may be a potential therapeutic target. Furthermore, ENY2 was found to facilitate breast cancer cell migration and metastasis both in vitro and in vivo [43]. This indicates that ENY2 is also an important molecule in tumor development and progression. As ENY-2 is known to be a Wnt inhibitory factor [44], it remains unclear whether impaired EYN-2 expression is associated with tumorigenesis through mRNP biogenesis. Direct evidence of the role of other components of the TREX-2 complex, namely centrin2/3 and DSS1, in tumorigenesis has not been reported.

## 7. Tumorigenesis in Deregulated Expression GANP

### 7.1. Aberrant Expression of GANP Protein in Human Tumors

The expression levels of *ganp* transcripts and GANP protein are extremely low in various tissues, except in normal mammary ducts. This evidence, together with GANP’s functions in DNA replication, suggests that GANP may be upregulated in cancer. Indeed, GANP upregulation was observed in various hematological disorders, including leukemias and lymphomas [45]. Aberrant GANP expression was also observed in malignant melanomas [46], liver fluke-associated cholangiocarcinomas [47], and testicular postpubertal-type teratomas [48]. Lymphomagenesis and teratomagenesis have also been linked to the abnormal expression of GANP [45,48]. In contrast, GANP expression was decreased in some solid tumors, such as in glioblastomas [49] and breast cancers [50]. The expression of GANP was also associated with resistance to breast cancer development. However, the level of GANP expression did not affect tumor migratory and invasive properties [50]. How GANP counteracts the oncogenic process in these tumors is yet to be elucidated. Using human diploid fibroblast MRC-5, the depletion of GANP showed the cell-cycle arrest of the cellular-senescence phenotype caused by the activation of p16 and the decrease in Rb expression [49]. These data indicated that the DNA-damage response caused by GANP insufficiency was mediated by the p16^INK4a^-Rb pathway; however, there is no evidence that GANP is directly associated with DNA repair pathway. A further study discovered an association between polymorphisms in the *GANP* locus, its expression, and breast cancer risk and prognosis [51]. These results suggest that GANP may contribute differently to tumorigenesis depending on various associated molecules or tissue type.

### 7.2. Lymphomagenesis in GANP Transgenic Mice

Further investigation into the mechanism underlying GANP’s involvement in lymphomagenesis has revealed its multifaceted impact on cellular processes. GANP, through its targeting of Lyn-mediated signaling within germinal center B cells, orchestrates a delicate balance crucial for cell survival and differentiation [52]. The PU.1 binding site in the *ganp* promoter serves as a key regulatory mechanism, allowing PU.1 to modulate the dynamic reprogramming of B cells and macrophage differentiation [53]. This regulatory network not only underscores the significance of GANP in maintaining the survival of mature germinal center B cells, but also highlights its role in suppressing DNA damage, thus safeguarding cellular integrity [54].

The abnormal overexpression of GANP has been documented in various human hematopoietic and lymphoid neoplasms, including Hodgkin and Reed–Sternberg (HRS) cells [45,55]. Furthermore, GANP is involved in the progression of phenotypic Hodgkinoid lymphoma, which displays features of B cells/macrophage phenotypic cells corresponding to human Hodgkin’s lymphoma.

GANP transgenic mice were generated under the immunoglobulin promoter and enhancer (*Ig*-*ganp*^Tg^). In *Ig*-*ganp*^Tg^ mice, B-cell/macrophage bi-phenotypic Hodgkinoid lymphoma developed, showing genetic fingerprint evidence of *Ig* gene rearrangement with expressions of Ig-μ/Ig-κ chains. GANP may also play a crucial role in ensuring the survival of HRS cells that stem from germinal center B cells in *Ig*-*ganp*^Tg^ mice [55]. These results of GANP’s role in lymphomagenesis suggests that its actions are not confined to individual cell types, but rather that it orchestrates a synergistic interplay between germinal center B cells and macrophages. The survival and transdifferentiation facilitated by GANP may serve as critical drivers of Hodgkin lymphomagenesis, shedding light on potential therapeutic targets for this complex malignancy [55]. The further elucidation of the molecular mechanisms underlying GANP’s function promises to deepen the understanding of lymphoma pathogenesis and may pave the way for more targeted therapeutic interventions.

### 7.3. Mammary Tumorigenesis in Ganp-Deficient Mice

**Figure 3:**
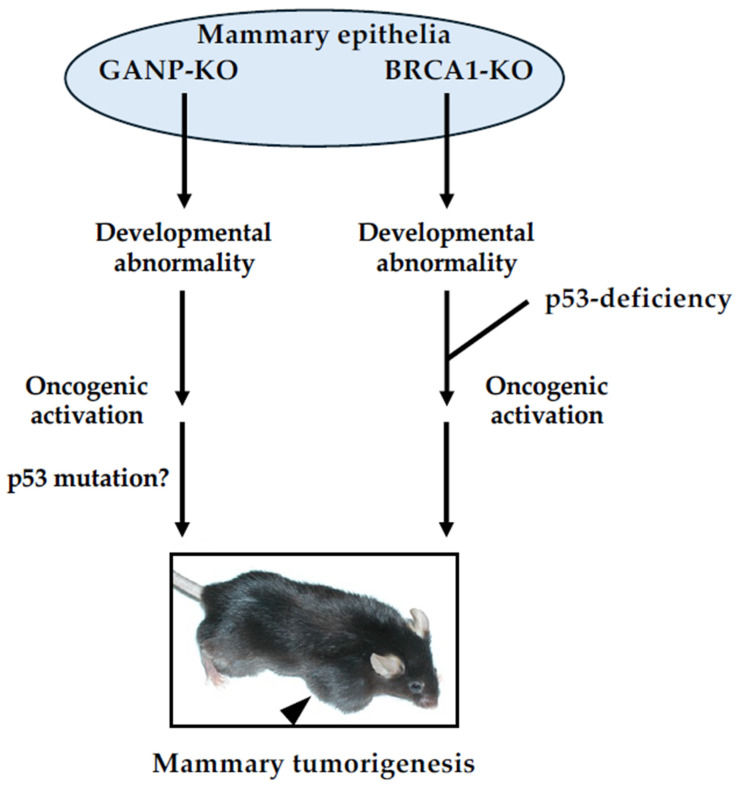
A model of BRCA1- or GANP-associated mammary tumorigenesis. Mammary-specific *brca1*-deletion results in the abnormal morphogenesis of mammary epithelia caused by growth arrest and cell death [56]. Mammary-specific *ganp*-homodeficient mice show a similar phenotype in the mammary development [50]. Several genetic alterations have accumulated in mammary glands under these abnormalities, leading to mammary tumorigenesis. In the case of *brca1*-deficient mice, mutations of p53 is related to growth advantage of tumor cells; however, the role of p53 in *ganp*-deficient mice remains unclear.

In contrast to GANP overexpression in hematological malignancies, GANP expression tended to decrease in the malignant progression of breast cancers. In a study of over 400 breast cancer patients, the GANP-low group showed a worse prognosis compared with the GANP-high group in both breast cancer-specific and relapse-free survival [50]. In normal mammary glands, GANP is highly expressed in the nuclei; GANP expression tended to downregulate in proportion to malignant progression. To examine the effects of downregulated GANP expression in vivo, a study was conducted by Kuwahara and colleagues to explore tumorigenesis in mammary glands with GANP deficiency. The deficiency in GANP expression in the mammary gland was constructed using mammary-specific *ganp*-deficient mice by crossing *ganp*-floxed mice with wap-cre mice. During pregnancy, cre recombinase is translated and cleaves inserted loxP sequences in the *ganp* gene, resulting in the induction of mammary-specific *ganp* deletion. The mammary-specific *ganp*-homodeficient mice were GANP-deficient, as confirmed by immunohistochemistry analysis and real-time polymerase chain reaction. During lactation, mice exhibited distinct malformations in mammary glands; the development of glandular anatomy was abnormal, the epithelial lining of the mammary gland lumen was misaligned, and the development of fibrous tissue was observed. After several gene alterations including mutations and translocations accumulated in mammary glands with abnormal morphology, mammary tumors developed. These phenotypes are similar to those observed in mammary-specific *brca1*-deficient mice [56] (Figure 3). The mammary-specific *ganp*-homodeficient mice also had metastases of tumor cells in the lung.

**Figure 4:**
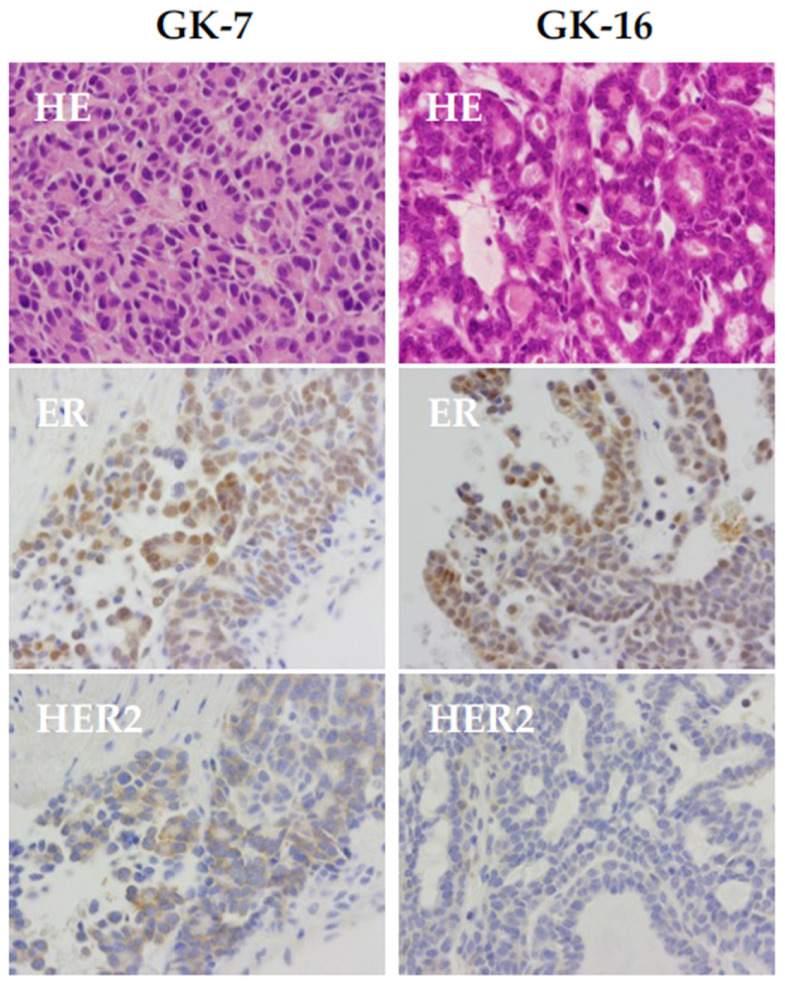
Aberrant expression of biomarkers related to human breast cancers in mammary tumor derived from aged *ganp*+/d mice. Most mammary tumors from genetically modified mice demonstrate a hormone receptor-negative phenotype, while mammary tumors occurred in *ganp*+/d mice show various phenotypes like luminal- and basal-like types in human breast cancers. Here, 70 to 80% of mammary tumors from *ganp*+/d mice are ER-positive, as shown [50]. Original magnification ×400 for each panel. GK-7 and GK-16 mean the *ganp*+/d mice numbers bearing mammary tumors. For details, please refer to Supplementary Materials.

**Figure 5:**
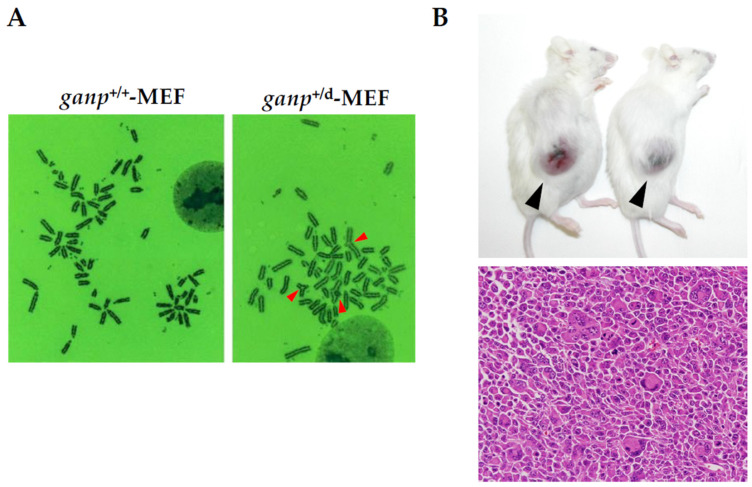
Oncogenic activity in *ganp*+/d-MEF. (**A**) Chromosomal analysis in *ganp*+/d-MEF demonstrate aberrant chromosomes like triradial chromosome indicated arrowheads. (**B**) After the inoculation of *ganp*+/d-MEF into Balb/c-Rag2/Jak3 double knockout mice [57], tumors developed as indicated by arrowheads (upper panel). Histological analysis showed sarcomatous tumors with bizarre nuclei (lower panel). Original magnification ×200. For details, please refer to Supplementary Materials.

Conventional *ganp*-homodeficient mice showed embryonic lethality [29]. The further investigation of *ganp*-heterodeficient (*ganp*^+/d^) mice confirmed that the expression of GANP was lower in comparison to the wild-type mice. The *ganp*^+/d^ mice during multiparity developed mammary gland tumors with the growth of atypical cells, suggesting that two alleles of *ganp* play a role in preventing mammary gland tumors. Tumors in *ganp*^+/d^ mice also displayed the aberrant expression of several biomarkers detected in human breast cancer, such as ERα, PgR, Her2, and Ki67 (Figure 4). Moreover, the analysis of cultured mammary gland tumor cells revealed aneuploidy as it was further confirmed in the longer duration of the culture exhibition of chromosomal instability at a high rate [50]. Mouse embryonic fibroblasts (MEFs) derived from *ganp*^+/d^ mice partially demonstrated triradial chromosomes, whereas *ganp*^+/+^-MEFs did not show any aberrant chromosomes (Figure 5A). Sarcomatous tumors with severe nuclear atypia were observed in immunodeficient mice inoculated with *ganp*^+/d^-MEFs [57], suggesting that cells deficient for GANP have oncogenic potential (Figure 5B). Moreover, to investigate the effect of GANP on estrogen-responding cells, DNA damage was measured in vitro in *ganp*-knockdown or *ganp*-overexpressed MCF7 cells after estrogen stimulation using the Comet assay to detect single-stranded and double-stranded DNA damage. Compared to control MCF7 cells, *ganp* depletion markedly augmented the estradiol-induced DNA damage, whereas *ganp*-transfected MCF7 cells had lower estradiol-induced DNA damage. In addition, the TUNEL assay also revealed that *ganp* depletion induces cell apoptosis [50]. These results indicate that GANP suppresses the DNA damage induced by estrogen exposure and has an anti-oncogenic effect on breast carcinogenesis.

One way that GANP may suppress tumors is through its interaction with DNA-dependent protein kinase, catalytic subunit (DNA-PKcs), which inhibits DSB repair by the non-homologous end-joining pathway and promotes repair by HR [54]. In addition to preventing tumor development, GANP is also required to maintain the lifespan of mice, as *ganp*^+/d^ mice were found to have a shorter lifespan than *ganp*^+/+^ mice.

## 8. Molecular Targets of Increased Chemosensitivity

Several components of the TREX-2 complex regulate the chemosensitivity of cancer cells. DSS1, a molecule of the TREX-2 complex that binds to BRCA2, has been associated with the occurrence of several types of tumors and sensitivity to chemotherapy drugs. In cervical cancer, DSS1 was reported as an upregulated molecule in cancerous lesions compared with normal cervixes [58]. A clinical study in female breast cancer patients revealed that those with elevated levels of DSS1 tended to experience poorer prognosis or shorter survival times compared with those with lower levels of DSS1 [59]. This may be caused by the interaction between DSS1 and the RPN3/S3 proteasomal subunit, leading to the enhanced degradation of ubiquitinated p53 [60]. A recent report suggested that DSS1 is critically involved in the proliferation, apoptosis, invasion, and migration of glioma cells via the Akt signaling pathway [61]. *dss1*-deficient mice have not yet been reported, and thus whether DSS1 is associated with tumorigenesis remains unclear. DSS1 depletion causes BRCA2 destabilization even in cells without BRCA1/2 germline mutation, leading to impaired HR repair in cancers (our unpublished results). Another study showed that DSS1 and PCID2 contribute to changes in the sensitivity threshold for anticancer drugs in vitro. PARPis may be effective in BRCA wild-type and DSS1-depleted breast cancer patients [5].

ENY-2, another component of the TREX-2 complex, may also be associated with decreased sensitivity to anticancer drugs, leading to chemoresistance. Proteomics was performed using the trastuzumab–pertuzumab-resistant HER-2^+^ breast cancer cell line. Among more than 600 proteins involved in important biological processes like metabolism, ribosome formation, and mitochondrial activity, ENY-2 was found to be upregulated or present in SK-BR3.rTP (resistant) cells, but not in SK-BR3 (sensitive) parental cells. The upregulation of ENY-2 could also contribute to the development of resistant HER-2^+^ breast cancer cells, although the detailed mechanism is still unclear [62]. As chemotherapy is often administered alongside other breast cancer treatments like surgery, radiation, or hormone therapy, these findings could be leveraged to develop additional chemotherapy preparation strategies, potentially enhancing clinical outcomes.

## 9. Conclusion and Perspective

The data concerning the relationship between the TREX-2 complex, especially GANP, and tumor occurrence and/or malignant progression from Section 5 to 8 is briefly summarized in Table 1. As described above, direct evidence of tumorigenesis caused by impaired TREX-2 expression using model mice is currently limited to GANP.

Transcription-coupled DNA damage has been extensively analyzed in *S. cerevisiae*. Recent evidence demonstrated that the deregulation of the mammalian TREX-2 component GANP affects tumorigenesis, especially lymphomagenesis and breast carcinogenesis [63]. Whether mRNA export per se is critical for carcinogenesis and/or cancer development remains to be elucidated. Germline mutations of *ganp* have not yet been identified in cancers. Nevertheless, GANP is an important gatekeeper to prevent the development of tumors. Mammary-specific *ganp*-deficient mice showed abnormal ductal development leading to the formation of mammary tumors, as observed in mammary-specific *brca1*-deficient mice [56]. The TREX-2 complex, including GANP, is functionally associated with BRCA2 [37]. GANP may be a BRCA-like molecule or function in concert with BRCA1 and/or BRCA2 in sporadic breast carcinogenesis (Figure 3).

Other TREX-2 components like PCID-2, ENY-2, and DSS1 are also related to tumorigenesis. The elevation of these proteins is associated with tumor progression, metastases, and chemoresistance, which is often associated with poor outcome in patients. Further studies are needed to elucidate the complex relationships between TREX-2 components and cancer in the hope that the results might identify a target for novel treatment that can save the lives of many patients.

## Figures and Tables

**Table 1:** Tumor occurrence and/or progression in deregulated TREX-2 expression.

TREX-2 Components	Expression of Human Tumors	Chemoresistance	Tumorigenesis in Mouse Model	References
GANP	Upregulation: Leukemia Lymphoma Melanoma Liver fluke-associated Cholangiocarcinoma Testicular teratoma Downregulation: Glioblastoma Breast cancer	ND	Gain-of-function: Lymphomagenesis Teratomagensis Loss-of-function: Mammary tumorigenesis	[45,46,47,48] [49,50]
PCID2	Upregulation: Colorectal cancer Promyelocytic leukemia	Upregulation Breast cancer	ND	[39,41]
DSS1	Upregulation: Cervical cancer Breast cancer	Upregulation Cervical cancer Breast cancer	ND	[58,59]
ENY2	Upregulation: Various cancers	Upregulation HER^2+^-breast cancer	ND	[42,43,62]

ND: not determined.

## Data Availability

The raw data supporting the conclusions of this article are available by the corresponding author (K.K.) on request.

## Supplementary Materials

The following supporting information can be downloaded at: https://www.mdpi.com/article/10.3390/ijms252413612/s1.
