# Supplementary material for: Tumorigenesis Caused by Aberrant Expression of GANP, a Central Component in the Mammalian TREX-2 Complex—Lessons from Transcription-Coupled DNA Damages
Source: Int J Mol Sci. 2024 Dec 19;25(24):13612. doi: 10.3390/ijms252413612 (PMC11727803; doi:10.3390/ijms252413612)
Supplement: Supplementary file 1 [file ijms-25-13612-s001.zip › ijms-3354509-supplementary.pdf]

Supplementary Materials and Methods

Mice. GANP-heterodeficient (*ganp*$^{+/d}$) mice were established previously [1]. Balb/c-Rag2/Jak3 double knockout mice were prepared as described [2]. All mice were maintained under specific-pathogen-free conditions in the animal facilities of the Graduate School of Kumamoto University and Aichi Cancer Center. All animal protocols were carried out following the approval of the Animal Care and Use Committee of the Graduate School of Medical Sciences of Kumamoto University and Aichi Cancer Center.

Histology. Mammary gland tumor samples and tumors excised from Balb/c-Rag2/Jak3 double knockout mice were fixed in 4% paraformaldehyde in PBS at 4 °C for 4 hours, embedded in paraffin, and cut into 4-μm-thick sections using a microtome. The sections were stained with hematoxylin and eosin according to the standard method. For IHC of mammary tumors from the *ganp* mutant mice, primary Abs against ERα (Abcam, Cambridge, UK) and Her2 (Agilent, Santa Clara, CA) were used in combination with a horseradish peroxidase-conjugated goat anti-rabbit IgG Ab (Thermo Fisher Scientific, Waltham, MA). Development was performed using 3,3-diaminobenzidine followed by counter-staining with hematoxylin.

Chromosome preparation. MEF cells were prepared as described previously [1]. Both *ganp*$^{+/+}$- and *ganp*$^{+/d}$-MEFs were cultured for 2 hours with nocodazole (100 ng/ml), harvested, suspended in 75 mM KCl, fixed in 3:1 methanol/glacial acetic acid, and air-dried on glass slides. Staining was performed using 5% Giemsa.

Generation of tumors derived from *ganp*$^{+/d}$-MEF in immunodeficient mice. Twenty million *ganp*$^{+/d}$-MEFs were inoculated into Balb/c-Rag2/Jak3 double knockout mice intraperitoneally. After two to three weeks, masses were excised for the further analysis.

Supplementary References

1. Yoshida, M.; Kuwahara, K.; Shimasaki, T.; Nakagata, N.; Matsuoka, M.; Sakaguchi, N. GANP Suppresses DNA Recombination, Measured by Direct-repeat *β-galactosidase* Gene Construct, but Does Not Suppress the Type of Recombination Applying to Immunoglobulin Genes in Mammalian Cells. *Genes Cells* **2007**, *12*, 1205–1213, doi: 10.1111/j.1365-2443.2007.01119.x.

2. Ono, A.; Hattori, S.; Kariya, R.; Iwanaga, S.; Taura, M.; Harada, H.; Suzu, S; Okada, S. Comparative Study of Human Hematopoietic Cell Engraftment into BALB/c and C57BL/6 Strain of Rag-2/Jak3 Double-deficient Mice. *J. Biomed. Biotechnol.* **2011**, *2011*, 539748, doi: 10.1155/2011/539748.
